# Free fatty acid-based low-impedance liver image: a characteristic appearance in nonalcoholic steatohepatitis (NASH)

**DOI:** 10.1186/s41747-019-0137-y

**Published:** 2020-01-23

**Authors:** Hitoshi Maruyama, Kazufumi Kobayashi, Soichiro Kiyono, Tetsuhiro Chiba, Naoya Kato, Masayuki Ohtsuka, Kazuyo Ito, Tadashi Yamaguchi, Shuichiro Shiina

**Affiliations:** 1 Department of Gastroenterology, Juntendo University, 2-1-1, Hongo, Bunkyo-ku, Tokyo, 113-8421 Japan; 2 Department of Gastroenterology, Chiba University Graduate School of Medicine, 1-8-1, Inohana, Chuo-ku, Chiba, 260-8670 Japan; 3 Department of General Surgery, Chiba University Graduate School of Medicine, 1-8-1, Inohana, Chuo-ku, Chiba, 260-8670 Japan; 4 Center for Frontier Medical Engineering, Chiba University, 1-33 Yayoicho, Inage, Chiba, 263-8522 Japan

**Keywords:** Acoustic impedance tests, Fatty acids, Liver, Nonalcoholic fatty liver disease, Ultrasonography

## Abstract

**Background:**

To examine *in vitro* acoustic property of nonalcoholic fatty disease in mouse and human liver to identify nonalcoholic steatohepatitis (NASH).

**Methods:**

The acoustic impedance (× 10^6^ kg/m^2^/s) was measured in 35 free fatty acids (FFAs, 500 mmol/L) and histologically-diagnosed liver samples of twelve mice (four control, four simple steatosis [SS], and four NASH) and eight humans (two control, three SS, and three NASH), using 80-MHz acoustic microscopy. The sum of percentage (SP) composition of FFAs (SP-FFAs) was also assessed.

**Results:**

Median impedance of all FFAs was 0.7 (5 FFAs with impedance 0.7); 17 FFAs with impedance < 0.7 were classified as low-impedance group; and, 13 FFAs with impedance > 0.7 were classified as high-impedance group. The median impedance of the mouse liver decreased from control (1.715), to SS (1.68), to NASH (1.635) (control *versus* NASH, *p* = 0.039 without significant differences for the other comparisons, *p* ≥ 0.1). Similarly, the median impedance of human liver showed decreased from control (1.825), to SS (1.788), to NASH (1.76) (control *versus* SS, *p* = 0.023; control *versus* NASH, *p* = 0.003; SS *versus* NASH, *p* = 0.050). The ratio of SP-FFAs between the low-impedance and high-impedance groups showed an increase in both mice and humans, with significant differences in mice (control *versus* SS, *p* < 0.001; control *versus* NASH, *p* < 0.001; SS *versus* NASH, *p* = 0.003), without significant differences in humans (*p* ≥ 0.671).

**Conclusion:**

Lower acoustic impedance based on the intrahepatic composition of FFAs may be characteristic of NASH.

## Key points

- The acoustic impedance differed according to the kinds of free fatty acids (FFAs).
- The acoustic impedance of mouse/human liver specimen showed gradual decrease from control, to simple steatosis, to nonalcoholic steatohepatitis (NASH).
- The acoustic impedance and percentage composition of intrahepatic FFAs may account for the pathophysiology of lower impedance in NASH.

## Background

Because of the increased incidence worldwide, nonalcoholic fatty liver disease (NAFLD) is considered one of the leading causes of chronic liver diseases [1]. Particularly, nonalcoholic steatohepatitis (NASH) is a progressive disease with potential risk for developing hepatocellular carcinoma (HCC) and portal hypertension [2, 3]. Investigators reported that early-stage NASH has a probability of 18–39% to progress to more advanced stages of hepatic fibrosis within 3.5–8.2 years [4–6]. An application of early liver biopsy is recommended, as an earlier intervention and more aggressive treatment may reduce overall mortality [6]. Thus, identification of NASH, particularly early-stage NASH, by effective imaging tools may be highly awaited in daily medical care.

Recent development of digital technologies has widened the application of novel imaging tools for NAFLD, such as the xenon computed tomography [7] and multi-scale electrical impedance tomography [8]. Against the background, ultrasound (US) is the most frequently used modality for the diagnosis of liver diseases because of the simplicity and non-invasiveness. Moreover, liver stiffness measurement and quantitative assessment of fat content using US technology has become available [9]. There are three types of US-based elastography, transient elastography, point share wave elastography, and two-dimensional share wave elastography. Because of the long-term experience and specific XL-probe for obese cases, numbers of evidence support the benefits of transient elastography with well-defined quality criteria [9]. Thus, transient elastography is recommended as a tool for staging hepatic fibrosis in the current guidelines on management of NAFLD [10].

However, despite of the convenience with the integrated setting in the US equipment, the scientific evidence in favor of point or two-dimensional share wave elastography is not enough and the usefulness in the obese case has not been clarified. Therefore, they are not included in the current guidelines [9, 10]. Also, as presented in a large cohort prospective study [11], diagnostic performance to assess liver steatosis and fibrosis using controlled attenuation parameter and liver stiffness measurements by FibroScan is not satisfactory. Differentiation between simple steatosis (SS) and NASH by US-based imaging alone is still under debate.

Since lower hepatic polyunsaturated fatty acids in NASH are reported to be associated with gene expression [12], non-invasive evaluation of hepatic free fatty acids (FFAs) may have a potential to characterize NAFLD. An animal study has shown that lower acoustic impedance may feature NASH and suggested that there was a difference in the acoustic property among five different kinds of FFAs [13]. However, it is not clear whether the human liver tissues show a similar result. Furthermore, the pathophysiology of lower impedance in NASH has not been investigated. Therefore, the present study prospectively examined the acoustic property of both mouse liver and surgically resected human liver samples. We also examined the pathogenesis of acoustic changes with respect to the impedance findings of FFAs which may differ in composition between SS and NASH.

## Methods

### Study outline

The study firstly examined the acoustic impedance of FFAs and liver samples of mice (control, SS, and NASH) and human subjects (control, SS, and NASH). Human liver samples of NASH were taken from the patients who underwent surgical treatment for HCC. To collect human liver samples with control or steatohepatitis, patients with metastatic liver tumor due to colon cancer were recruited.

Secondly, the effect of percentage composition of intrahepatic FFAs on the impedance of liver sample was examined, and finally, the relationship between the impedance of the liver and the histological findings was assessed. The animal/clinical study was approved by the ethical committee of Chiba University (27-113, 339), and informed written consent was obtained from all participants.

### Sample preparation

#### Free fatty acids

The study used 35 kinds of FFAs (Table 1). Samples of FFAs for measurements were made at final concentrations of 500 mmol/L, in accordance with the literature [14].

**Table 1:** Impedance in free fatty acids

Acid	Chemical name	Mean	Standard deviation
Butyric acid	4:0	0.71	0.01
Caproic acid	6:0	0.72	0.01
Caprylic acid	8:0	0.71	0.01
Capric acid	10:0	0.69	0.01
Undecanoic acid	11:0	0.72	0.01
Lauric acid	12:0	0.67	0.01
Tridecanoic acid	13:0	0.71	0.01
Myristic acid	14:0	0.7	0.01
Myristoleic acid	14:1	0.65	0.01
Pentadecanoic acid	15:0	0.69	0.01
Palmitic acid	16:0	0.68	0.01
Palmitoleic acid	16:1 (n-7)	0.49	0.01
Heptadecanoic acid	17:0	0.7	0.01
Stearic acid	18:0	0.71	0.01
Oleic acid	18:1 (n-9)	0.72	0.01
Elaidic acid	18:1 (n-9t)	0.69	0.01
Linoleic acid	18:2 (n-6)	0.66	0.01
Linolelaidic acid	18:2 (n-6t)	0.71	0.01
γ-Linolenic acid	18:3 (n-6)	0.69	0.01
α-Linolenic acid	18:3 (n-3)	0.53	0.01
Arachidic acid	20:0	0.7	0.01
cis-11-Eicosenoic acid	20:1	0.72	0.01
cis-11,14-Eicosadienoic acid	20:2	0.72	0.01
cis-8,11,14-Eicosatrienoic acid	20:3 (n-6)	0.7	0.01
cis-11,14,17-Eicosatrienoic acid	20:3 (n-3)	0.71	0.01
Arachidonic acid	20:4 (n-6)	0.6	0.01
cis-5,8,11,14,17-Eicosapentaenoic acid	20:5 (n-3)	0.69	0.01
Heneicosanoic acid	21:0	0.71	0.01
Behenic acid	22:0	0.69	0.01
Erucic acid	22:1 (n-9)	0.7	0.01
cis-13,16-Docosadienoic acid	22:2	0.72	0.01
cis-4,7,10,13,16,19-Docosahexanoic acid	22:6 (n-3)	0.68	0.01
Tricosanoic acid	23:0	0.53	0.01
Lignoceric acid	24:0	0.68	0.01
Nervonic acid	24:1	0.65	0.01

#### Animal liver sample

The study used 6- to 10-week-old male STAM^TM^ mice, which were purchased from Stelic Institute & Co. (Charles River Laboratories, Japan, Inc.). The mouse model was established by the following protocol according to the literature [15]; 2-day-old male pups were injected with streptozotocin (200 μg per mouse) and started on a high-fat diet (HFD-32) from the age of 4 weeks. The animals developed steatosis to steatohepatitis from 6 to 8 weeks of age and fibrosis from 9 to 12 weeks of age, showing various grades of NAFLD. The study also used control mice on a control diet. All mice were euthanized by the inhalation of methoxyflurane to collect the blood sample and sacrificed to obtain liver samples, which were immediately rinsed in saline to remove blood and cut into approximately 1-mm-thick slices. Liver sample was divided into three blocks and used for (i) immediate measurement of acoustic impedance, (ii) Masson trichrome staining, and (iii) kept frozen in liquid nitrogen for FFA analysis. The microscopic findings, including a presence/absence or degree of fibrosis or steatosis, were confirmed histologically according to the criteria reported in the previous study [16].

#### Human liver sample

Non-tumor liver samples of participants were carefully taken using surgically resected specimens. The sample was divided into three blocks (approximately 1 cm^3^ for each block) for (i) immediate measurement of acoustic impedance, (ii) Masson trichrome staining, and (iii) kept frozen in liquid nitrogen for FFA analysis. For impedance measurement, liver samples were immediately rinsed in saline to remove blood and cut into approximately 1-mm-thick slices. The microscopic findings, including a presence/absence or degree of fibrosis or steatosis, were confirmed histologically according to the criteria reported in a previous study [16].

### Extraction of FFAs

Lipids were extracted from liver tissue (approximately 100 mg per mouse/human) according to Folch’s method with chloroform/methanol [17]. Total fatty acid content (free and esterified, μg/g) in the liver tissue was measured by gas chromatography (chromatography profiles) with the samples prepared by chloroform and methanol using GC-2010 Plus (Shimadzu, Kyoto, Japan).

### Impedance measurement

Scanning acoustic microscopy was performed using modified AMS-50SI (Honda Denshi, 2.4 mm × 2.4 mm, point 300 × 300, average 8, range 200 mV, applied voltage 21.5 V [80 MHz]). Each FFA and fresh liver specimen on the polystyrene substrate was scanned to provide acoustic impedance (× 10^6^ kg/m^2^/s) according to the literature [13]. The sum of percentage composition of each FFA in the liver was compared with respect to the degree of the impedance level.

### Statistical analysis

Parametric data are reported as mean ± standard deviation (SD), while non-parametric data are reported as median and interquartile range (IQR). Normality of continuous variables was confirmed by using Shapiro-Wilk test, and the data were compared by Student’s *t* test or Fisher’s protected least significant difference test. Probability values lower than 0.05 were considered to be statistically significant. The statistical values were calculated using the SAS software (SAS Institute, Inc., Cary, NC, USA).

## Results

### Acoustic impedance analysis of FFAs

The median value of the impedance (× 10^6^ kg/m^2^/s) of all FFAs was 0.7 (range, 0.49–0.72; mean ± SD, 0.68 ± 0.056) (Table 1). The impedance of five FFAs was 0.7. In the remaining 30 FFAs, 17 FFAs with impedance lower than 0.7 were classified as the low-impedance group, and 13 FFAs with impedance higher than 0.7 were classified as the high-impedance group.

### Acoustic impedance analysis of mouse liver

#### Liver tissue

**Fig. 1:**
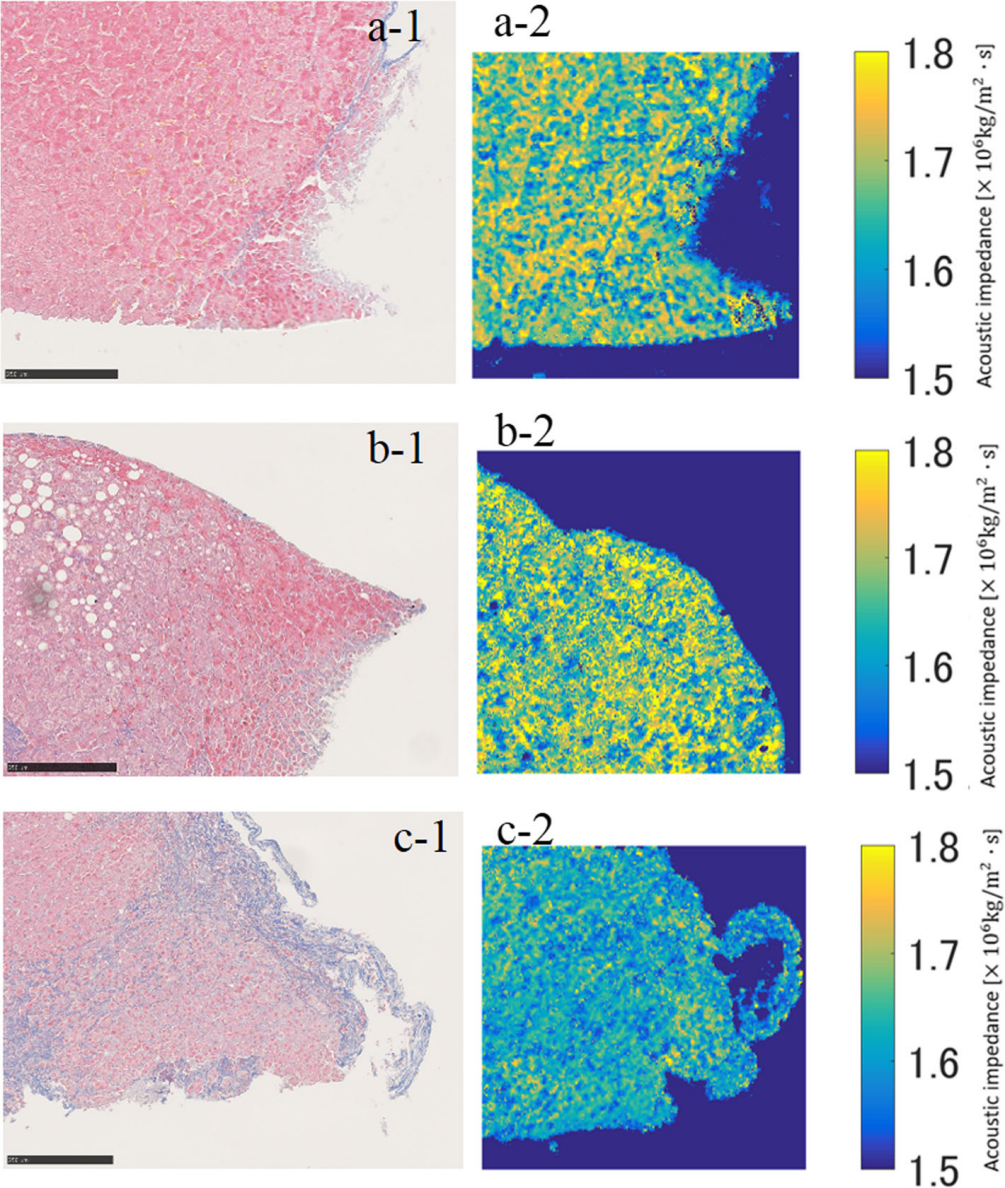
Images of mouse liver sample. **a** Control. (**a-1**) Masson trichrome stain of a control: no fibrosis and no steatosis; (**a-2**) acoustic impedance image: there is a nearly even distribution of yellow area and blue/green area; median acoustic impedance 1.74 × 10^6^ kg/m^2^/s. **b** Simple steatosis. (**b-1**) Masson trichrome stain: no fibrosis with 40% fat deposition; (**b-2**) acoustic impedance image: the blue/green area is larger than the yellow area; median acoustic impedance 1.66 × 10^6^ kg/m^2^/s. **c** Nonalcoholic steatohepatitis. (**c-1**) Masson trichrome stain: advanced fibrosis (F3 or F4) with 30% fat deposition; (**c-2**) acoustic impedance image: the blue/green area is dominant; median acoustic impedance 1.6 × 10^6^ kg/m^2^/s

There were twelve mice in the study (Table 2), four in the control group, four in the SS group, and four in the NASH group (Fig. 1). The impedance (× 10^6^ kg/m^2^/s) of the liver showed a gradual decrease from control (median 1.715, IQR 0.06), SS (median 1.68, IQR 0.05), to NASH (median 1.635, IQR 0.025), showing differences between control and SS (*p* = 0.554), control and NASH (*p* = 0.039), and SS and NASH (*p* = 0.113).

**Table 2:** Characteristics in mouse model

	Week	Body weight (g)	Fat	Fibrosis	Impedance
Control	10	30	–	F0	1.75 ± 0.05
Control	6	11	–	F0	1.74 ± 0.13
Control	9	25	–	F0	1.64 ± 0.13
Control	8	26	–	F0	1.69 ± 0.08
SS	6	9	20%	F0	1.66 ± 0.16
SS	6	8	30%	F0	1.66 ± 0.15
SS	6	9	40%	F0	1.70 ± 0.11
SS	6	10	40%	F0	1.74 ± 0.06
NASH	8	17.3	40%	F1-2	1.67 ± 0.06
NASH	8	19.1	40%	F1-2	1.60 ± 0.02
NASH	10	20	20%	F3-4	1.64 ± 0.02
NASH	8	20	30%	F3-4	1.63 ± 0.11

Impedance (× 10^6^ kg/m^2^/s), mean, and standard deviation

*NASH* Nonalcoholic steatohepatitis, *SS* Simple steatosis

#### FFAs in the liver

**Fig. 2:**
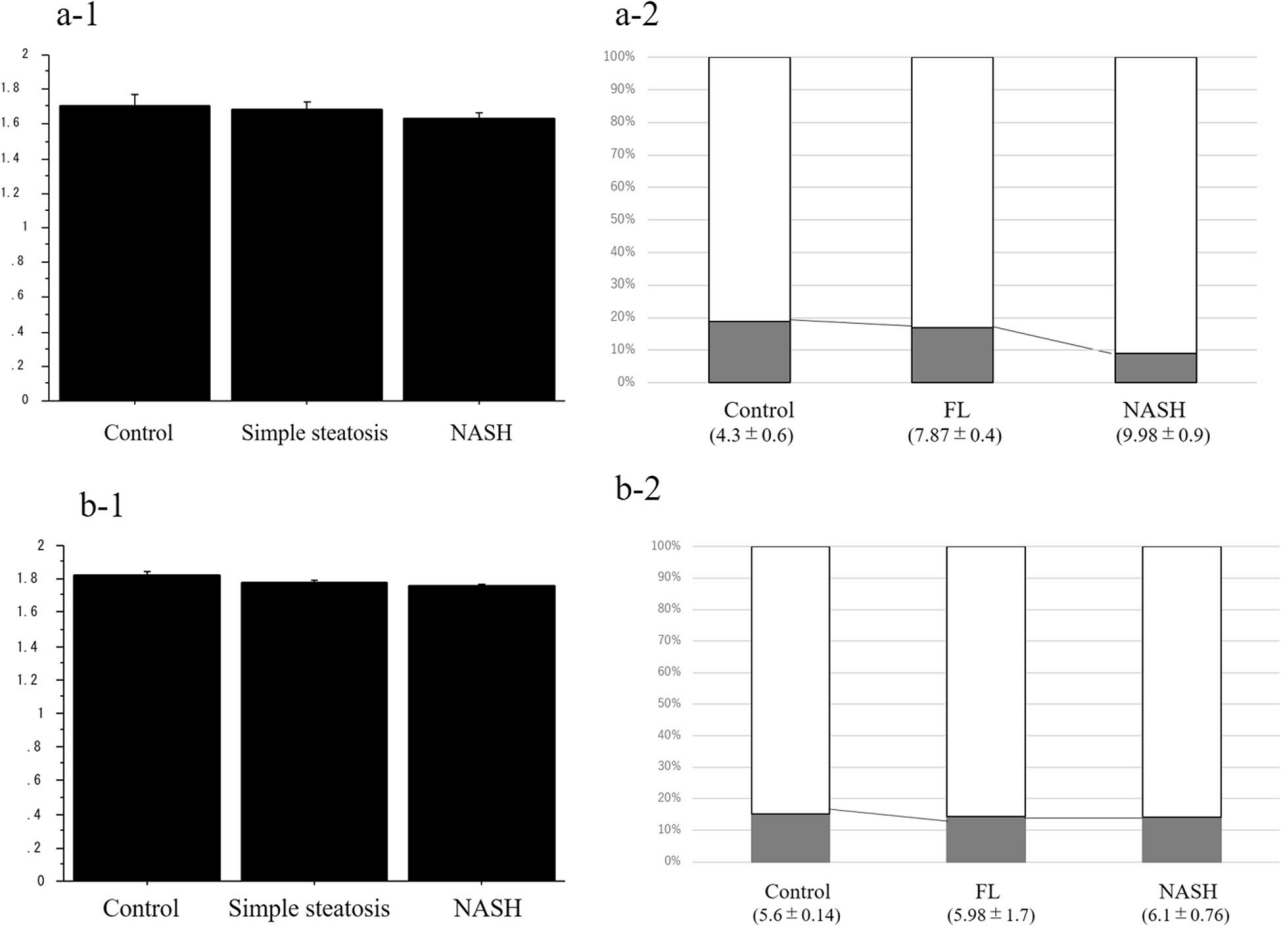
Acoustic impedance analysis. **a** Mouse. (**a-1**) Liver sample: the acoustic impedance (× 10^6^ kg/m^2^/s) showed a gradual decrease from control (median 1.715, IQR 0.06), SS (median 1.68, IQR 0.05), to NASH (median 1.635, IQR 0.025), showing differences between control and SS (*p* = 0.554), control and NASH (*p* = 0.039), and SS and NASH (*p* = 0.113). (**a-2**) FFA in the liver: the ratio of sum of percentage composition of FFAs between the low-impedance group and the high-impedance group showed gradual increases from control (median 4.45, IQR 0.65; 3.58–4.87), SS (median 7.92, IQR 0.322; 7.23–8.39), to NASH (median 9.9, IQR 0.73; 8.94–11.2), with significant differences between control and SS (*p* = 0.0002), control and NASH (*p* < 0.001), and SS and NASH (*p* = 0.003). White, low-impedance group; gray, high-impedance group. Data are presented as mean and standard deviation. **b** Human liver sample. (**b-1**) Liver sample: the acoustic impedance (× 10^6^ kg/m^2^/s) showed gradual decrease from control (median 1.825, IQR 0.015), SS (median 1.788, IQR 0.0045), to NASH (median 1.76, IQR 0.015), showing differences between control and SS (*p* = 0.023), control and NASH (*p* = 0.003), and SS and NASH (*p* = 0.050). (**b-2**) Free fatty acids in the liver: the ratio of sum of percentage composition of FFAs between the low-impedance group and the high-impedance group showed a gradual increase from control (median 5.7, IQR 0.012; 5.53–5.73), SS (median 5.9, IQR 0.0029; 4.23–7.75), and NASH (median 6.2, IQR 0.0021; 5.3–6.8). However, there were no significant differences between control and SS (*p* = 0.758), control and NASH (*p* = 0.671), and SS and NASH (*p* = 0.893). White, low-impedance group; gray, high-impedance group. Data are presented as mean and standard deviation. *FFA* Free fatty acid, *IQR* Interquartile range, *NASH* Nonalcoholic steatohepatitis, *SS* Simple steatosis

A concentration (mg/g) of intrahepatic FFAs is summarized in Table 3. The ratio of sum of percentage composition of FFAs between the low-impedance and the high-impedance groups showed gradual increases from control (median 4.45, IQR 0.65; 3.58–4.87), SS (median 7.92, IQR 0.322; 7.23–8.39), to NASH (median 9.9, IQR 0.73; 8.94–11.2), with significant differences between control and SS (*p* < 0.001), control and NASH (*p* < 0.001), and SS and NASH (*p* = 0.003) (Fig. 2).

**Table 3:** Concentration of free fatty acids in mouse liver sample

	1	2	3	4	5	6	7	8	9	10	11	12
	Contr.	Contr.	Contr.	Contr.	SS	SS	SS	SS	NASH	NASH	NASH	NASH
C14:0	LLOQ	LLOQ	LLOQ	LLOQ	0.1	0.2	0.2	0.1	0.1	0.1	0.4	0.2
C15:0	LLOQ	LLOQ	LLOQ	LLOQ	LLOQ	0.1	0.1	LLOQ	LLOQ	0.1	LLOQ	0.1
C16:0	4.9	4.1	6.0	4.7	7.2	10.5	9.1	8.5	10.2	11.8	11.8	13.8
C16:1	0.2	0.2	0.4	0.2	0.1	0.2	0.4	0.1	0.2	2.2	0.4	1.3
C17:0	LLOQ	0.1	0.1	LLOQ	0.3	0.3	0.2	0.3	0.4	0.4	0.6	0.4
C18:0	2.8	2.3	3.2	2.8	3.9	4.4	3.3	3.6	1.0	2.1	1.6	1.8
C18:1n9c	2.2	1.9	3.5	2.0	21.5	29.5	31.9	20.7	27.1	31.0	57.0	37.4
C18:2n6c	4.9	3.6	4.6	3.7	4.4	7.0	4.4	5.1	3.8	2.5	1.4	4.5
C18:3n6	LLOQ	LLOQ	LLOQ	LLOQ	0.1	0.4	0.1	0.2	0.2	0.2	0.6	0.3
C18:3n3	LLOQ	LLOQ	0.1	LLOQ	LLOQ	LLOQ	LLOQ	LLOQ	LLOQ	LLOQ	LLOQ	LLOQ
C20:1n9	LLOQ	LLOQ	0.1	LLOQ	0.5	0.3	0.9	0.3	0.3	0.4	0.3	0.5
C20:3n6	0.4	0.3	0.5	0.3	0.5	0.4	0.4	0.4	0.5	0.6	0.5	0.8
C20:4n6	2.2	2.2	3.9	2.9	3.6	3.6	3.5	3.0	4.2	4.1	4.9	4.5
C20:5n3	0.6	0.4	LLOQ	LLOQ	LLOQ	LLOQ	LLOQ	LLOQ	LLOQ	LLOQ	LLOQ	LLOQ
C22:6n3	3.5	2.6	2.7	2.8	3.2	3.6	2.3	3.8	1.8	2.0	1.3	2.0

Data are presented as concentration (mg/g)

*Contr.* Control, *LLOQ* Lower limit of quantification, *SS* Simple steatosis, *NASH* Nonalcoholic steatohepatitis

FFAs unlisted in the table were LLOQ

### Acoustic impedance analysis of human liver

#### Liver tissue

**Fig. 3:**
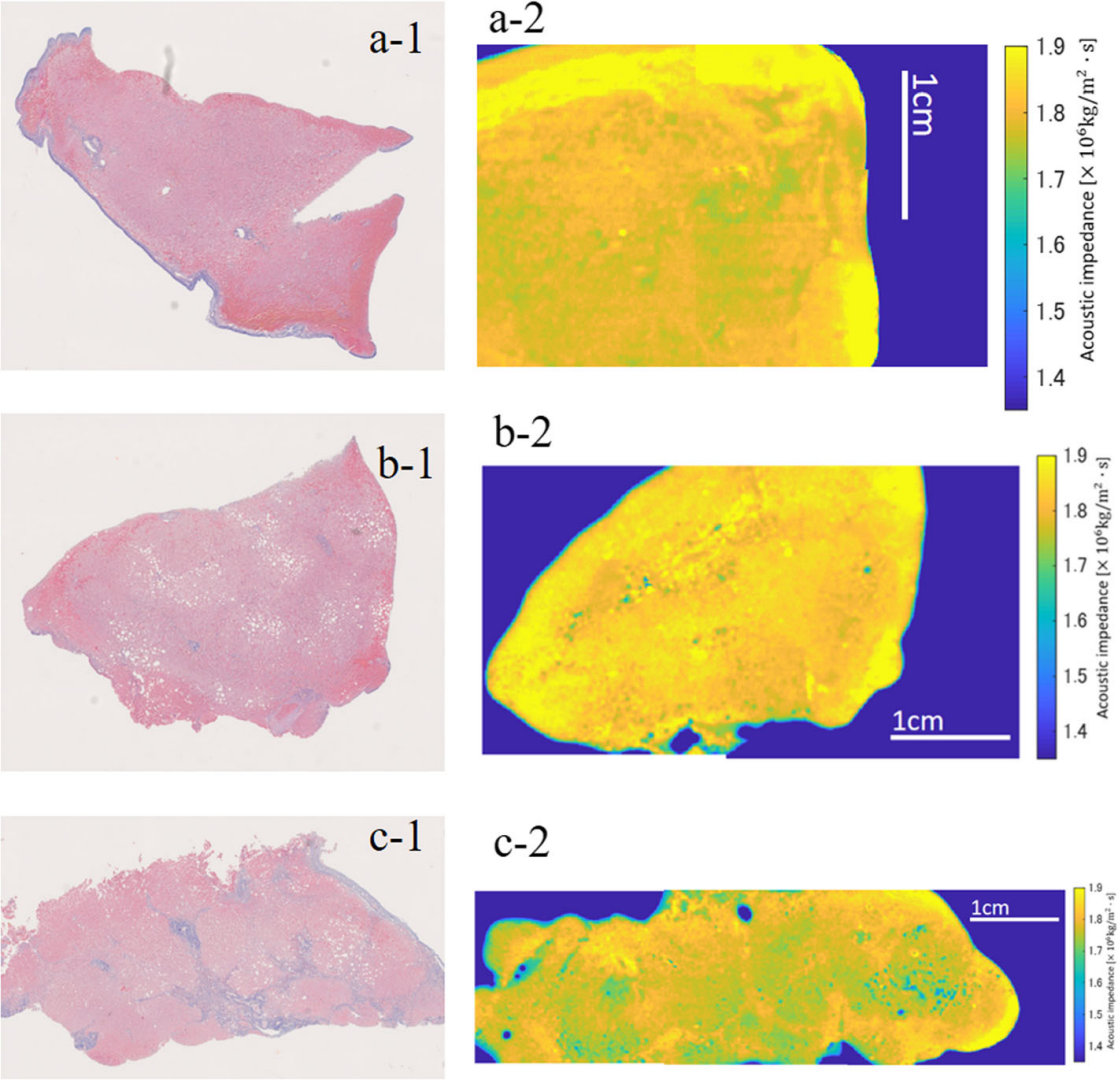
Images of human liver sample. **a** Control. (**a-1**) Masson trichrome stain: no fibrosis and no steatosis. (**a-2**) Acoustic impedance image: light yellow area and dark yellow area with a few green part; median acoustic impedance was 1.81 × 10^6^ kg/m^2^/s. **b** Simple steatosis. (**b-1**) Masson trichrome stain: no fibrosis with 40% fat deposition. (**b-2**) Acoustic impedance image: light yellow area and dark yellow area with a few green part; median acoustic impedance was 1.78 × 10^6^ kg/m^2^/s. **c** Nonalcoholic steatohepatitis. (**c-1**) Masson trichrome stain: advanced fibrosis (F3-4) with 20% fat deposition. (**c-2**) Acoustic impedance image: dark yellow area and green area are dominant; median acoustic impedance was 1.77 × 10^6^ kg/m^2^/s

There were eight patients in the study (Table 4), two in the control group, three in the SS group, and three in the NASH group (Fig. 3). The impedance (× 10^6^ kg/m^2^/s) of the liver also showed gradual decrease from control (median 1.825, IQR 0.015), SS (median 1.788, IQR 0.005), to NASH (median 1.76, IQR 0.015), showing differences between control and SS (*p* = 0.023), control and NASH (*p* = 0.003), and SS and NASH (*p* = 0.050).

**Table 4:** Characteristics of human subjects

	Age	Sex	LD	Fat	Fibrosis	ALT	T-BIL	ALB	PT	PLT	Impedance
						(U/L)	(mg/dL)	(g/dL)	(%)	(10^4^/μL)	× 10^6^ kg/m^2^/s
1	76	M	–	0	0	24	0.5	4.3	123	24	1.84 ± 0.01
2	76	M	-	0	0	14	0.7	3.8	104	13.4	1.81 ± 0.01
3	76	F	SS	40%	0	20	0.9	4.5	105	21.6	1.79 ± 0.01
4	54	M	SS	5%	0	18	0.6	4.5	98	36.1	1.79 ± 0.02
5	75	M	SS	20%	0	47	1.2	4	95	11.1	1.78 ± 0.014
6	75	M	NASH	50%	F4	16	0.8	3.3	87	11.6	1.74 ± 0.015
7	74	M	NASH	30%	F1	25	1.2	3.6	96	20	1.76 ± 0.01
8	77	M	NASH	40%	F4	14	1.6	3.5	85	13.4	1.77 ± 0.02

*ALB* Albumin, *ALT* Alanine aminotransferase, *F* Female, *LD* Liver disease, *M* Male, *NASH* Nonalcoholic steatohepatitis, *PLT* Platelet count, *PT* Prothrombin time, *SS* Simple steatosis, *T-BIL* Total bilirubin

#### FFAs in the liver

Concentration (mg/g) of intrahepatic FFAs is summarized in Table 5. The ratio of the sum of percentage composition of FFAs between the low-impedance group and the high-impedance group showed a gradual increase from control (median 5.7, IQR 0.012; 5.53–5.73), SS (median 5.9, IQR 0.003; 4.23–7.75), and NASH (median 6.2, IQR 0.002; median IQR; 5.3–6.8) (Fig. 2). However, there were no significant differences between control and SS (*p* = 0.758), control and NASH (*p* = 0.671), and SS and NASH (*p* = 0.893).

**Table 5:** Concentration of free fatty acid in human liver sample

		Contr.	Contr.	SS	SS	SS	NASH	NASH	NASH
C16:0		7.6	5.6	6.4	8.1	2.8	1.3	2.3	1.0
C18:1n9c		5.5	5.5	5.5	2.0	1.6	1.3	1.3	1.7
C18:2n6c		4.6	3.7	4.9	3.8	1.9	2.0	1.2	2.2
C18:0		2.8	2.0	2.4	3.3	1.6	3.1	1.1	2.8
C16:1		0.5	0.7	0.4	1.2	0.1	1.3	1.9	1.1
C22:6n3		1.9	1.6	2.2	2.1	0.5	1.1	2.2	1.2
C14:0		0.2	0.2	0.1	0.8	LLOQ	0.4	0.6	0.5
C20:4n6		1.7	1.2	1.5	2.9	1.3	1.9	2.5	1.0
C18:3n3		0.1	0.1	0.3	0.7	LLOQ	1.9	1.4	0.4
C20:5n3		0.3	0.2	0.3	0.2	LLOQ	0.1	0.3	0.3
C17:0		0.1	LLOQ	LLOQ	0.3	LLOQ	LLOQ	0.2	0.1
C20:3n6		0.2	0.1	0.1	0.3	0.1	0.2	0.1	0.1
C20:1n9		LLOQ	0.1	LLOQ	LLOQ	LLOQ	LLOQ	0.1	0.1
C15:0		LLOQ	LLOQ	LLOQ	LLOQ	LLOQ	LLOQ	0.1	LLOQ
C22:0		LLOQ	LLOQ	LLOQ	0.1	LLOQ	LLOQ	LLOQ	LLOQ

Data are presented as concentration (mg/g)

*Contr.* Control, *LLOQ* Lower limit of quantification, *NASH* Nonalcoholic steatohepatitis, *SS* Simple steatosis; FFAs unlisted in the table were LLOQ

## Discussion

Non-invasive characterization of NAFLD has been a clinical requirement. A demonstration of key factors to identify NASH is a considerable issue in the creation of a novel imaging tool which enables early and definite diagnosis. To the best of our knowledge, this is the first study to report the characteristics of the acoustic properties in NASH livers. The NAFLD liver samples in both mouse and human subjects are related with lower acoustic impedance, and it tended to decrease according to the disease progression, from SS to NASH. The authors stress that this unique feature should represent the potential to develop a radiological imaging alternative to liver biopsy.

Although a previous study has shown that the impedance differs depending on the kind of FFAs, the data in the study were obtained with only five FFAs, linoleate acid, α-linolenic acid, oleate acid, palmitate acid, and palmitoleic acid [13]. The present study, with the use of 35 FFAs, further demonstrated that the impedance varies according to the kind of FFA. Moreover, by the interpretation of intrahepatic percentage composition of FFAs, the impedance of FFAs may account for the pathophysiology of lower impedance in NASH. As previously reported, there is a difference in the plasma phospholipid and FFA composition between SS and NASH [18, 19]. However, there is no relationship in the FFA composition between liver tissue and serum [18, 20], and this fact may enhance the application of radiological imaging based on intrahepatic FFAs. Identification of factors that specify the acoustic properties of FFAs may be challenging in the future.

The intrahepatic FFA composition differs between control, SS, and NASH [20]. In the present study, pentadecanoic acid, palmitic acid, palmitoleic acid, elaidic acid, and linoleic acid in the low-impedance group were elevated, and stearic acid in the high-impedance group was decreased in NASH livers. The data may be compatible with the previous report [20] and may explain the lower acoustic impedance in NASH. However, contrary to our results, the remaining six FFAs in the low-impedance group (α-linolenic acid, arachidonic acid, nervonic acid, cis-4,7,10,13,16,19-docosahexanoic acid, γ-linolenic acid, and cis-5,8,11,14,17-eicosapentaenoic acid) were reported to be decreased, and two FFAs in the high-impedance group (oleic acid and linolelaidic acid) were reported to be increased in NASH livers [20]. The differences in race between Asian and Western countries may be one of the reasons for the results. Moreover, interaction of different kinds of FFAs in the liver may affect the mutual acoustic characteristics, which needs to be determined in the future.

One of the typical imaging modalities based on acoustic parameters is ultrasound elastography, an assessment tool using propagation velocity, which has attracted interest worldwide. A recent study has shown increased velocity with the degree of hepatic fibrosis and decreased velocity with the accumulation of fat [21]. However, the early stage of NASH shows less fibrosis, and the assessment of fat deposition may not be effective to differentiate between SS and NASH, suggesting the difficulty in the early diagnosis of NASH. In fact, a study performed in 164 biopsy-proven NAFLD patients [22] has shown that vibration-controlled TE could rule out advanced fibrosis and avoid the need for biopsy in at least 45% patients with NAFLD in the USA. A more well-designed prospective study reported that the model with both the liver stiffness measurement and controlled attenuation parameter had an area under the receiver operating characteristic of 0.71 in diagnosing NASH, which appears unsatisfactory [23]. Taken together, current US-based quantitative tools do not seem sufficiently sensitive to identify steatohepatitis without advanced fibrosis in patients with NAFLD. It is expected that an FFA-based impedance technique may overcome this problem because composition of FFAs shows characteristic features even in pre-cirrhotic NASH livers.

A recent study [21] reported the higher impedance in fibrotic livers than in normal livers. This finding may be reasonable because the presence of fibrosis may resist sound wave propagation. Our study demonstrated that the difference of the acoustic impedance between SS and NASH was not statistically significant (*p* = 0.113) in mice, and that was marginal (*p* = 0.050) in human. The data may be explained by the influence of the presence of fibrosis on the acoustic impedance as a confounding factor. It should be further investigated whether the interrelationship between fibrosis and fat may affect the acoustic data.

The major limitation of our study is that the data are based on measurements using an 80-MHz transducer using much higher frequencies than those typically used in the clinical setting. Second, the observation setting may also be far from that in the human body, which shows much greater attenuation affected by the physical size and intervening tissues. Thirdly, small sample size, particularly in human subjects, may limit the value of the data. The reason is that the acoustic measurement required surgically resected specimens because percutaneous biopsy samples were too small to perform the measurement. Such obstacles must be overcome for the impedance-based system to be equipped in the actual US machine.

In conclusion, this study identified lower acoustic impedance in NAFLD, with the reduction appearing dominant in NASH livers. The acoustic property may be based on the intrahepatic composition of FFAs showing characteristic impedance. These data strongly encourage the practical application of this technique to identify NASH in the near future.

## Data Availability

The datasets used and/or analyzed during the current study are available from the corresponding author on reasonable request.

## Abbreviations

FFA: Free fatty acid
HCC: Hepatocellular carcinoma
IQR: Interquartile range
NAFLD: Nonalcoholic fatty liver disease
NASH: Nonalcoholic steatohepatitis
SD: Standard deviation
SS: Simple steatosis
US: Ultrasound
